# The relation between sensory processing sensitivity and telomere length in adolescents

**DOI:** 10.1002/brb3.2751

**Published:** 2022-08-29

**Authors:** Armin Jentsch, Frances Hoferichter, Diana Raufelder, Geja Hageman, Lou Maas

**Affiliations:** ^1^ University of Greifswald Department of Education Greifswald Germany; ^2^ Maastricht University Department of Pharmacology & Toxicology, Nutrim, Research Institute of Nutrition and Translational Research in Metabolism Maastricht The Netherlands

**Keywords:** adolescents, perceived stress, sensory processing sensitivity, telomere length

## Abstract

**Background:**

In the present study, we investigated the association between sensory processing sensitivity (SPS) and telomere length (TL), which is considered a biomarker of cellular aging. SPS is an individual characteristic describing increased perception and procession of inner or outer stimuli, and is positively related to self‐perceived stress.

**Methods:**

We recruited 82 healthy adolescents aged 13–16 from secondary schools in Germany. SPS was measured with the Highly Sensitive Person Scale, and TL was determined by a multiplex quantitative PCR method.

**Results:**

Our results show that students with higher values of SPS are likely to have shorter telomeres (*β* = 0.337, *p* = .001), when adjusting for sex, socioeconomic status, age, and body mass index. These findings are also independent of the negative impact of stress students might have perceived shortly before data collection.

**Conclusions:**

Our analysis suggests that students who struggle with low sensory threshold are likely to have shorter telomeres.

## Graphical abstract

In the present study, we investigated into the association between sensory processing sensitivity (SPS) and telomere length, which is considered a biomarker of cellular aging. Our results show that students with higher values of SPS are likely to have shorter telomeres. These findings are also independent from the negative impact of stress students might have perceived shortly before data collection.

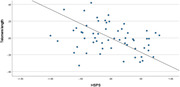

## 1 INTRODUCTION

In their seminal work, Aron and Aron (1997) introduced the concept of sensory processing sensitivity (SPS) which is an individual characteristic describing increased perception and procession of inner or outer stimuli. They found that—although related—SPS was not to be confused with other personality traits like introversion or neuroticism (see also Konrad & Herzberg, 2017). Kagan et al. (1994) propose that about 15%–20% of the population could be regarded as highly sensitive. It has been shown that a highly sensitive person (HSP) is more likely to perceive negative affectivity (Aron et al., 2005), anxiety (Neal et al., 2002), stress and burnout (Hoferichter & Raufelder, 2020), and symptoms of illness than fellow individuals (Benham, 2006). Thus, SPS could ultimately be related to serious health problems caused by chronic stress (Konrad & Herzberg, 2017).

Telomeres are repeated DNA sequences located at the ends of chromosomes, and their length reduces naturally over time (Mather et al., 2011). This decrease in telomere length is the consequence of incomplete synthesis of telomere sequences in newly formed DNA strands during cell replication, or to the induction of damage to telomeric DNA, which is less well repaired and enhances telomere shortening (Rosiello et al., 2022). However, if individuals are exposed to high levels of stress, maintenance of telomere length might be compromised and the shortening process might accelerate (Lin & Epel, 2022). Thus, telomere length (TL) is regarded as a biomarker of cellular aging (Blackburn, 2005) and predicts severe diseases and mortality (Farzaneh‐Far et al., 2008; Rentscher et al., 2020). Furthermore, there is evidence that TL could be associated with personality traits like pessimism and neuroticism (O'Donovan et al., 2009; van Ockenburg et al., 2014).

However, there is still limited empirical evidence on whether SPS contributes to biological aging over and above self‐perceived stress, both in general and in specific populations, including school students. Therefore, we investigate the association between high sensitivity and TL in the present study. It is hypothesized that healthy, more sensitive adolescents tend to have shorter telomeres than their peers.

## 2 METHODS

### 2.1 Measures

#### 2.1.1 Telomere length

Saliva sampling (Oragene•DNA (OG‐500)) and DNA extraction (prepIT•L2P | PT‐L2P) kits were used to collect and store saliva, and extract DNA, following the manufacturer's instructions (DNA Genotek®, Steinbrenner Laborsysteme GmbH, Germany). Following extractions, quality and concentration of the extracted DNA were determined using a NanoDrop spectrophotometer (Isogen Life Science, Belgium). Absorbance at 260 and 280 nm was measured, and extracted DNA was found to be of good quality with A260/280 between 1.7 and 2.1. TL was measured as previously described (Gielen et al., 2014). In brief, TL was determined using a monochrome multiplex quantitative PCR (q‐PCR) method, using 384 multiwell plates (Roche, Switzerland) that were run on a LightCycler 480 machine (Cawthon, 2009). Reference samples with known TL, that is, 5.5 kB (Hela S3 cell line) and 14.5 kB (Hela 229 cell line), were included into each run to enable estimation of TL in kB. Samples were measured in triplicate and the average was used.^1^

#### 2.1.2 Sensory processing sensitivity

The Highly Sensitive Person Scale (HSPS) was originally developed by Aron and Aron (1997) as a unidimensional score to measure SPS. The HSPS in its current version uses 15 items that are answered on a five‐point scale (1: fully disagree, 5: fully agree). Cronbach's α for the total HSPS score was 0.79, which is regarded as good internal consistency. More recent work has also revealed that three subscales of the HSPS can be distinguished (Konrad & Herzberg, 2017), namely ease of excitation (EOE, eight items, e.g., “I startle easily,” *α* = 0.72), aesthetic sensitivity (AES, four items, e.g., “I am deeply moved by music,” *α* = 0.55), and low sensory threshold (LST, three items, e.g., “I become unpleasantly aroused when a lit is going on around me,” *α* = 0.73), yielding more information than a unidimensional score. Thus, we consider both the effects of the total HSPS score and three subscale scores on TL in the present study.

#### 2.1.3 Perceived stress

The Perceived Stress Scale (PSS‐10) is a 10‐item version of one of the most widely used measures to capture acute distress and coping abilities (S. Cohen et al., 1983; Kechter et al., 2019). The PSS‐10 refers to stress experiences in the last month (e.g., “I felt difficulties were piling up so high that I could not overcome them”), and the answers were given on five‐point scales (1: never, 5: often). There is some debate among researchers about whether the PSS‐10 is better represented by two correlated factors than a unidimensional score, with strong arguments for both sides (for an overview see Lee, 2012). However, since perceived stress is not under focus in the present study and our sample size is small, we decided to use the PSS‐10 total score, for which internal consistency was very good (Cronbach's *α* = 0.92).

#### 2.1.4 Control variables

Evidence suggests that TL could systematically differ by age, body mass index (BMI), sex, and socioeconomic status (Rentscher et al., 2020). To avoid confounding of model results, we therefore controlled for the corresponding variables, including a dummy variable for sex (0: female, 1: male), and the number of books available in the participants’ households as a proxy for socioeconomic status (0: less than 100 books, 1: more than or equal to 100 books, see, e.g., Avvisati, 2020).

### 2.2 Participants and procedure

The present study is part of a larger project in which a total of 733 seventh‐ and eighth‐grade students from 11 randomly selected public secondary schools and 60 classrooms in the federal state of Mecklenburg‐Western Pomerania (Germany) participated. Before data collection, approval from the Ministry of Education, Science and Culture of Mecklenburg‐Western Pomerania, the data protection officer, and the ethics committee of the university medical center was obtained. Subsequently, the consent forms from the participating students and their parents were obtained. We recruited a randomly selected subsample of 82 healthy, unrelated adolescents from secondary schools in the federal state of Mecklenburg‐Vorpommern, Germany. The survey was conducted in the classrooms at school and the saliva collection in the rooms of the university, before samples were analyzed in the laboratory. Relevant sample characteristics are presented in Table 1. Approximately half of the students were female, and they were aged 13–16. More than three quarters had a high socioeconomic status (i.e., more than 100 books in their households). Average height and weight were 1.70 ± 0.08 m or 62.2 ± 15.2 kg, respectively. The resulting body mass indices showed that 18 students were underweight (BMI <18 kg/m^2^), 48 had normal weight (BMI = 18–24.9 kg/m^2^), 11 were overweight (BMI = 25–30 kg/m^2^), and four were considered obese (BMI > 30 kg/m^2^) involving one missing value.

**TABLE 1:** Sample characteristics (*n* = 82)

Sex female/male	40/42
SES low/high	18/64
Age (years)	
mean ± SD (range)	13.7 ± 0.7 (13–16)
BMI (kg/m^2^)	
Mean ± SD (range)	21.4 ± 4.3 (12.8–33.5)
PSS‐10 (1–5)	
Total score mean ± SD	2.8 ± 0.8
HSPS (1–5)	
Total score mean ± SD	2.7 ± 0.5
EOE mean ± SD	2.7 ± 0.7
AES mean ± SD	3.2 ± 0.7
LST mean ± SD	2.2 ± 0.7
TL (kb)	
mean ± SD (range)	12.03 ± 2.43 (8.4–20.7)

Abbreviations: AES, aesthetic sensitivity; BMI, body mass index; EOE, ease of excitation; HSPS, Highly Sensitive Person Scale; LST, low sensory threshold; PSS‐10, perceived stress scale; SES, socioeconomic status; SD, standard deviation; TL, telomere length.

### 2.3 Statistical analysis

Analysis was performed with IBM SPSS 25.0. We considered statistical significance at *p* < .05 and used J. Cohen's (1992) classification for effect sizes. TL was transformed by natural logarithm (mean transformed TL was 2.47 ± 0.19) and inspected for normality, both visually and by conducting a Kolmogorov–Smirnov test. To answer our research questions, we first calculated bivariate Pearson correlations for all study variables and then performed multiple regression analysis with TL as a dependent variable. We controlled for sex, socioeconomic status, age, BMI, and explored the additive effects of high sensitivity (HSPS) over and above perceived stress (PSS‐10). To avoid multicollinearity issues, we considered separate models for the total HSPS score and three subscale scores EOE, AES, as well as LST. Clustering of the data (i.e., students within schools) was taken into account by estimating robust standard errors for the regression coefficients.

## 3 RESULTS

### 3.1 Bivariate correlations

TL was normally distributed (*p* = .097) after excluding one outlier. Table 2 presents bivariate Pearson correlations with estimated *p*‐values for all study variables. Note that the results for the overall HSPS score were very similar to those of the three subscales (Pearson correlation with *p*‐values for sex: *r* = −0.25, *p* = .026, SES: *r* = 0.27, *p* = .015, age: *r* = −0.10, *p* = .459, BMI: *r* = −0.17, *p* = .131, PSS‐10: *r* = 0.53, *p* = .001, TL: *r* = −0.32, *p* = .004). Female students and those with higher socioeconomic status tended to be more sensitive, but the effect size was small.

**TABLE 2:** Bivariate Pearson correlations for all variables (top row, *n* = 82) with estimated *p*‐values (bottom row)

	(1)	(2)	(3)	(4)	(5)	(6)	(7)	(8)
1. Sex	–							
2. SES	−0.327	–						
	0.003							
3. Age	0.210	−0.054	–					
	0.101	0.677						
4. BMI	0.102	−0.006	0.241	–				
	0.365	0.958	0.061					
5. PSS‐10	−0.177	0.097	−0.157	0.040	–			
	0.112	0.391	0.224	0.721				
6. EOE	−0.139	0.189	−0.033	−0.108	0.570	–		
	0.213	0.091	0.798	0.338	0.001			
7. AES	−0.234	0.195	−0.176	−0.112	0.315	0.305	–	
	0.035	0.083	0.175	0.322	0.004	0.006		
8. LST	−0.283	0.280	−0.083	−0.230	0.161	0.496	0.343	–
	0.010	0.011	0.522	0.039	0.148	0.001	0.002	
9. TL	0.120	−0.021	0.119	0.159	−0.213	−0.334	−0.152	−0.201
	0.292	0.857	0.370	0.166	0.060	0.003	0.183	0.075

Abbreviations: AES, aesthetic sensitivity; BMI, body mass index; EOE, ease of excitation; LST, low sensory threshold; PSS‐10, perceived stress scale; SES, socioeconomic status; TL, telomere length.

### 3.2 Multiple regression analysis

**FIGURE 1:**
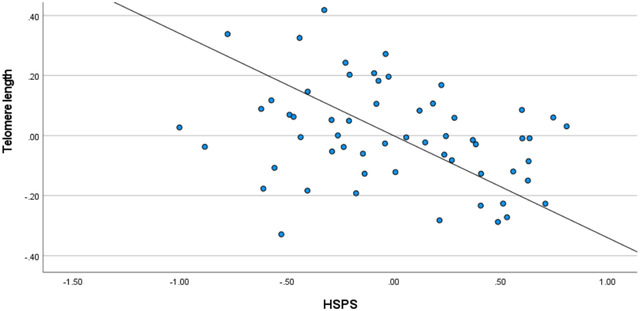
Relationship between standardized Highly Sensitive Person Scale (HSPS) scores and standardized telomere length (transformed by natural logarithm)

Table 3 presents the results of multiple regression analyses. Perceived stress was negatively associated with TL. Effect sizes ranged from small (*β* = −0.17, model 3) to moderate (*β* = −0.37, model 1). High sensitivity was also negatively associated with TL (HSPS total score: *β* = −0.34, model 2 and Figure 1). Subscale analysis revealed that only LST was negatively associated with TL (*β* = −0.44, moderate effect size, model 3). For ease of excitation and aesthetic sensitivity, no statistically significant effects were found. Note that in all presented models, we controlled for sex, socioeconomic status, age, and BMI. However, none of these effects were statistically significant. To ensure the robustness of our results, we also estimated a baseline model in which TL was regressed on the control variables only (not presented here). Similarly, no statistically significant effects were found.

**TABLE 3:** Effects of the Perceived Stress Scale (PSS‐10) and Highly Sensitive Person Scale (HSPS) on telomere length (*n* = 82)

	Model 1		Model 2		Model 3	
Variables	*β*	*p*‐Value	*β*	*p*‐Value	*β*	*p*‐Value
PSS‐10	−0.371	0.002	−0.209	0.013	−0.191	0.022
HSPS			−0.337	0.001		
EOE					−0.111	0.329
AES					0.178	0.120
LST					−0.437	0.010
*R* ^2^	0.180		0.258		0.338	

*Notes*: The dependent variable is telomere length (transformed by natural logarithm). All models are controlled for sex, socioeconomic status, age, and BMI.

Abbreviations: AES, aesthetic sensitivity; EOE, ease of excitation; LST, low sensory threshold.

## 4 DISCUSSION

The present study focused on the relation between sensory processing sensitivity and cellular aging in adolescents. We found a negative association of up to moderate size for both the HSPS total score and the LST subscale score. This suggests that students who struggle with low sensory threshold are likely to have shorter telomeres. Because we controlled for perceived stress in our analysis, the findings are independent of the negative impact of stressful situations students might have experienced shortly before data collection.

In a similar vein, Benham (2006) showed that SPS was related to symptoms of illness over and above self‐perceived stress. However, in the subscale analysis, Ahadi and Bashapoor (2010) found that not only LST but also EOE was related to relevant psychological outcomes (e.g., depression and anxiety). This could be due to the sample size, but also to the fact that EOE is more proximal with regard to mental health, rather than general health outcomes such as TL. Several underlying psychological mechanisms could thus explain the observed correlations. On one hand, SPS and TL are both related to personality traits such as neuroticism (e.g., Konrad & Herzberg, 2017; van Ockenburg et al., 2014). On the other hand, high sensitivity could be associated with health‐harming behavior, which in turn is related to telomere shortening (Mather et al., 2011; Suzuki et al., 2017).

Our study is not without limitations. First, the AES subscale suffered from low reliability that might have influenced the effect sizes in our findings. Adequate statistical methodology such as latent variable modeling is available to deal with measurement error (e.g., Jöreskog, 1971). However, this was not feasible in this study due to small sample size. Second, it should be noted that our sample consisted only of students aged 13–16 from a specific area in Germany. This suggests that it might be worthwhile to replicate our findings in different ethnic groups or countries. Third, this study reported results from a cross‐sectional study, making it impossible to investigate causal relationships between the study variables. Therefore, future research could focus more on the longitudinal development of TL across the life span, which Boonekamp et al. (2014) have already pointed out. They present evidence that *telomere shortening* might be an even more accurate biomarker of biological aging processes than TL. The reason for this is that TL is considered an outcome of both genetic and developmental influences, which suggests confounding of different processes or variables. Therefore, it might also be promising to investigate the effects of protective factors that are associated with decreased telomere shortening in future studies (e.g., parental support, Brody et al., 2015; Hoferichter et al., 2021).

## CONFLICT OF INTEREST

The authors declare no conflict of interest.

### PEER REVIEW

The peer review history for this article is available at https://publons.com/publon/10.1002/brb3.2751.

## Data Availability

The data that support the findings of this study are available on request from the corresponding author. The data are not publicly available due to privacy or ethical restrictions.

## ENDNOTE

1 For multiplex q‐PCR, telomere primer pairs telg (at 900 nm; ACACTAAGGTTTGGGTTTGGGTTTGGGTTTGGGTTAGTGT) and telc (at 900 nm; TGTTAGGTATCCCTATCCCTATCCCTATCCCTATCCCTAACA) were combined with the beta‐globin primer pairs hbgu (at 500 mM; CGGCGGCGGGCGGCGCGGGCTGGGCGGcttcatccacgttcaccttg) and hbgd (at 500 mM; GCCCGGCCCGCCGCGCCCGTCCCGCCGgaggagaagtctgccgtt).
